# Second Attack of Scarlet Fever

**Published:** 1914-11

**Authors:** 


**Second Attack of Scarlet Fever.** Lammerhirt, "Progressive Med.," Jan. 1914, reports three cases in an experience of 10 years, averaging 30-50 cases a year. The intervals were 18 months, 6 years, 8 months, in children respectively 3½, 11, and 12, at the second attack. (Note: None of the semelincident diseases are absolutely so, although recurrences are rare. There are no accurate statistics available, largely because the history is liable to confuse exanthemata. As the microrganism of most of the common semelincident infections is unknown, and diagnosis rests merely on clinical appearances, it is obvious that a further factor of uncertainty is introduced. Indeed, as a possibility, we may even go to the extreme of raising the question as to whether the exanthemata and certain other infections, apparently distinct are not modified manifestations of one or a few true specific infections. Ed.)

